# Fe(NO_3_)_3_-assisted large-scale synthesis of Si_3_N_4_ nanobelts from quartz and graphite by carbothermal reduction–nitridation and their photoluminescence properties

**DOI:** 10.1038/srep08998

**Published:** 2015-03-11

**Authors:** Shuyue Liu, Minghao Fang, Zhaohui Huang, Juntong Huang, Haipeng Ji, Haitao Liu, Yan-gai Liu, Xiaowen Wu

**Affiliations:** 1 School of Materials Science and Technology, Beijing Key Laboratory of Materials Utilization of Nonmetallic Minerals and Solid Wastes, National Laboratory of Mineral Materials, China University of Geosciences, Beijing, 100083; 2 College of Engineering, Mathematics and Physical Sciences, University of Exeter, Exeter EX4 4QF, UK

## Abstract

The large-scale synthesis of Si_3_N_4_ nanobelts from quartz and graphite on a graphite-felt substrate was successfully achieved by catalyst-assisted carbothermal reduction–nitridation. The phase composition, morphology, and microstructure of Si_3_N_4_ nanobelts were investigated by X-ray diffraction, Fourier transform infrared spectroscopy, field-emission scanning electron microscopy, energy-dispersive spectroscopy, transmission electron microscopy, and high-resolution transmission electron microscopy. The Si_3_N_4_ nanobelts were ~4–5 mm long and ~60 nm thick and exhibited smooth surfaces and flexible shapes. The Si_3_N_4_ nanobelts were well crystallized and grow along the [101] direction. The growth is dominated by the combined mechanisms of vapor–liquid–solid base growth and vapor–solid tip growth. The Fe(NO_3_)_3_ played a crucial role in promoting the nanobelt formation in the initial stage. The room-temperature photoluminescence spectrum of Si_3_N_4_ nanobelts consists of three emission peaks centered at 413, 437, and 462 nm, indicating potential applications in optoelectronic nanodevices.

One-dimensional (1D) nanostructures (e.g., nanowires, nanotubes, and nanobelts) have attracted considerable attention owing to their unique morphologies, excellent properties, and potential applications in nano-optoelectronic devices and nanocomposites^1^,^2^,^3^,^4^,^5^. Among the various nanostructures, nanobelts with the special shape of a rectangle-like cross-section and high surface areas have attracted much interest, and many types of nanobelts in different material systems have been successfully synthesized^6^,^7^,^8^. Silicon nitride (Si_3_N_4_) is well known for its excellent mechanical properties and thermal stability; it is one of the most important high-temperature structural ceramics. Moreover, a wide band gap (5.0–5.3 eV) of Si_3_N_4_ makes it possible to tailor its electro-optic properties by proper doping^9^. Because of these features, Si_3_N_4_ is a promising material for optical and electronic devices operating in harsh environments such as high temperatures and radiation environments.

To date, Si_3_N_4_ nanobelts have been successfully synthesized by several methods. Yang et al. synthesized Si_3_N_4_ nanobelts by the catalyst-assisted (e.g., FeCl_2_, NiCl_2_, and Al powder) pyrolysis of polysilazane under a flow of N_2_ or NH_3_^10^,^11^,^12^. Huo et al. reported the direct nitridation of Fe–Si alloy particles^13^. Yin et al. reported the vapor–solid reaction of NH_3_ with SiO^14^. Although many of these methods produced high-yield Si_3_N_4_ nanobelts, some difficulties were encountered. For instance, it is difficult to separate high-quality nanobelts from the remaining powder in vapor–solid reactions. For the pyrolysis, the liquid polysilazane has to be solidified by heat treatment before the pyrolysis, and the residual carbon derived from the reaction still remains in the product. Huang et al.^15^ separated the starting raw materials from the catalytic substrate. This modified thermal chemical vapor deposition (CVD) method overcame these difficulties in separation/purification. Previous studies focused on the successful synthesis of high-purity products with different reaction mechanisms. In all these methods, chemical reagents such as Si powder, SiO powder, and polysilazane have been selected as the raw materials. Although high-purity reagents can improve the quality of products, the purification of raw materials increases the cost of production. Therefore, it is important to develop an efficient, low cost, large-scale method for the synthesis of Si_3_N_4_ nanobelts. Carbothermal reduction–nitridation (CRN) is a common method for the synthesis of inorganic powder materials, but it has been rarely used to synthesize 1D nanomaterials.

In this paper, quartz and graphite, which are abundant in nature and are easy to acquire, were selected as the raw materials to prepare Si_3_N_4_ nanobelts by the CRN method. Moreover, the modified CVD route was used to simplify the product separation process. A method that covers both sides of the graphite-felt substrate with Fe(NO_3_)_3_ catalyst increased the nucleating points and improved the yield of product. The as-fabricated Si_3_N_4_ nanobelts were characterized, and the growth mechanism was elucidated. Furthermore, the room-temperature photoluminescence (PL) performance of Si_3_N_4_ nanobelts was measured at the UV light excitation.

## Results

### Phase, morphology, and microstructure of Si_3_N_4_ nanobelts

**Figure 1:**
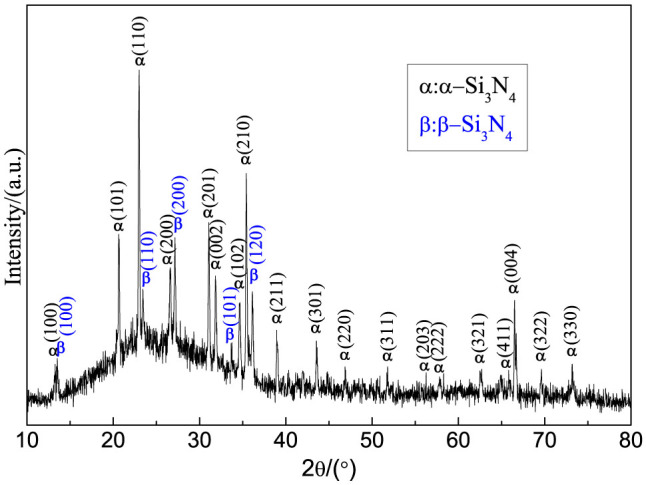
XRD pattern of the as-synthesized products.

**Figure 2:**
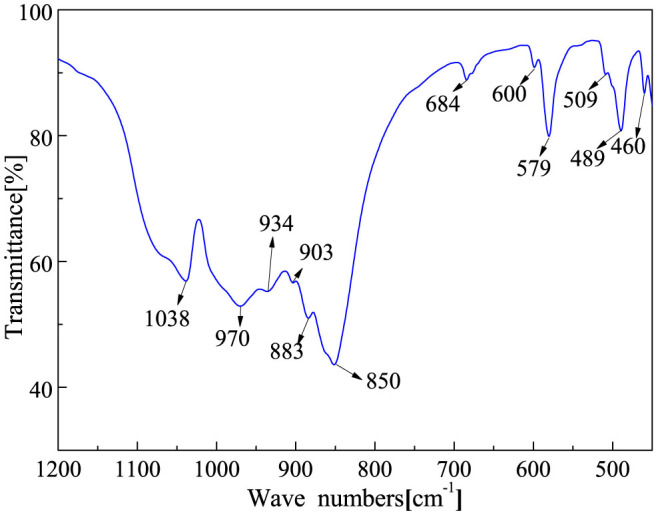
FT-IR spectrum of the products detached from the graphite felt.

After removing from the vacuum furnace, a layer of white-color product was observed on the upper side of the graphite-felt substrate, which was designed on the top of the graphite crucible as a substrate. When carefully lifted with tweezers, the underside of the substrate was also covered with similar product. For further characterization, the synthesized product was peeled from the graphite-felt substrate. Compared to the conventional methods where the raw material was mixed with the catalyst, the separation of product and raw material simplified the purification procedure. The X-ray diffraction (XRD) pattern of the product (Fig. 1) showed that both the α-Si_3_N_4_ and β-Si_3_N_4_ were present in the cotton-like product. As shown in the XRD pattern, all the lines of α-Si_3_N_4_ from 10 to 80° could be indexed to α-Si_3_N_4_ (JCPDS Card No. 76-1407), whereas the main lines of β-Si_3_N_4_ were identified at the lower-angle regions (JCPDS Card No. 82-698). A broad bulge between 15° and 35° originated from the glass substrate used to load the product. The phase composition of Si_3_N_4_ was further confirmed by FT-IR spectrum as shown in Fig. 2. A series of absorption peaks from 800 to 1100 cm^−1^ can be attributed to the Si–N–Si skeletal vibration of Si_3_N_4_^16^,^17^. Some unbiased testimony can be obtained from the region 450–700 cm^−1^, known as the fingerprint region of an IR spectrum. Representative peaks were observed at 684, 600, and 460 cm^−1^, because of the formation of α-Si_3_N_4_ phase^18^,^19^. Simultaneously, a sharp peak at 579 cm^−1^ can be attributed to the difference between α- and β-Si_3_N_4_, which was used to quantitatively determine the amount of the two types of Si_3_N_4_^20^.

**Figure 3:**
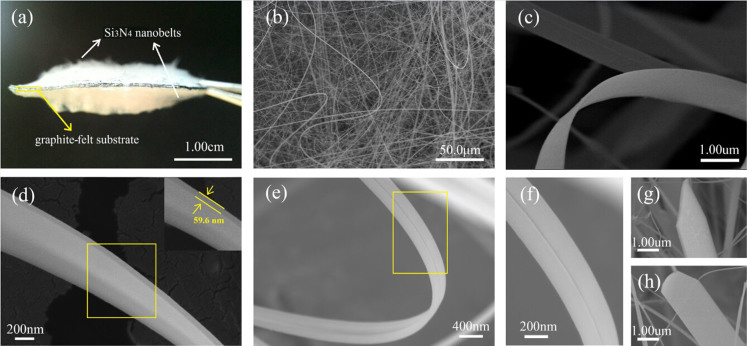
(a) Digital photograph and (b–h) SEM imagesof the as-synthesized Si_3_N_4_ nanobelts.

Fig. 3a shows the image of large amounts of white-color product, visibly grown on the graphite-felt substrate. As shown in the picture, both sides of the felt were fully covered by cotton-like fiber product, because both sides of the substrate were daubed by Fe(NO_3_)_3_ catalyst. The thickness of the upper side layer was ~4 mm, whereas the thickness of the underside layer was ~5 mm, probably caused by different concentrations of reaction gas during the heating process. The length of the Si_3_N_4_ nanobelts can reach as long as several millimeters. In contrast to the previous one-side covering of catalyst^21^,^22^, the yield of the product obtained by this method clearly increased. The morphology of the Si_3_N_4_ product was further investigated by scanning electron microscopy (SEM). The low-magnification SEM image shown in Fig. 3b shows that the as-synthesized Si_3_N_4_ has an unordered 1D structure. The curved and screwy shape indicated that the 1D Si_3_N_4_ nanobelts are flexible and elastic. The high-magnification SEM image shown in Fig. 3c indicates that this 1D structure was almost belt-like. The thickness of a typical nanobelt was ~60 nm (Fig. 3d). Moreover, one type of special structure was found on the surface of some nanobelts as shown in Figs. 3e and 3f. A clear straight trail crossed the nanobelt and seemed to divide the entire part into two parts; this is discussed in detail later. The tip of the 1D structure has attracted much attention because it demonstrates the growth mechanism of the nanostructured materials. Figs. 3g and 3h show the triangular tips of Si_3_N_4_ nanobelts, and no spherical particles were observed.

**Figure 4:**
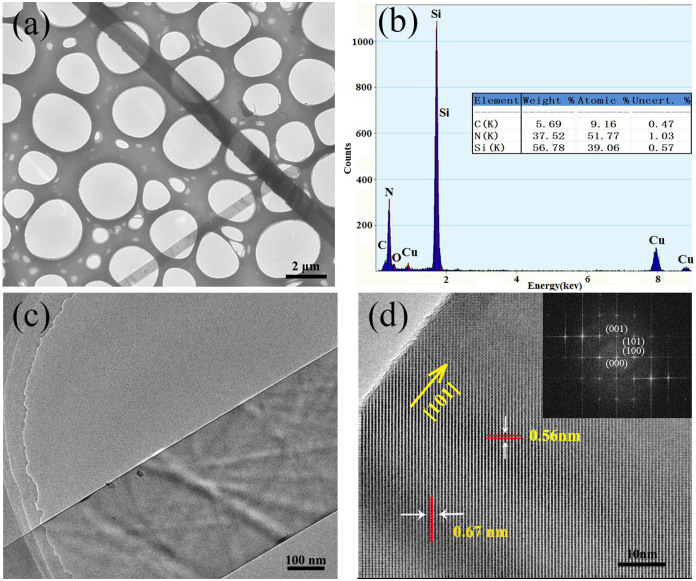
(a) TEM image. (b) EDS pattern of α-Si_3_N_4_ nanobelts. (c) High magnification TEM of an individual α-Si_3_N_4_ nanobelt. (d) High resolution TEM image and fast Fourier transformation (FFT) image (insert) of the α-Si_3_N_4_ nanobelt.

The crystal structure of the Si_3_N_4_ nanobelts was further investigated by transmission electron microscopy (TEM), high-resolution TEM (HRTEM), and energy-dispersive spectroscopy (EDS). Figs. 4a and 4c show that the width of nanobelts along the length direction is consistent. The EDS result shows that the nanobelts contain Si and N in the atomic ratio of 0.754:1, close to the stoichiometric ratio in Si_3_N_4_ (0.750:1) (Fig. 4b). Minor C and O peaks appeared due to a porous carbon membrane on the copper grid and raw quartz supplies. A typical HRTEM image of a single Si_3_N_4_ nanobelt is shown in Fig. 4d. The clear lattice fringes show that the nanobelts were well crystallized. Moreover, lattice-fringe spacings of 0.67 nm and 0.56 nm conformed well to the (001) and (100) planes of α-Si_3_N_4_, respectively. The inset of Fig. 4d shows the corresponding selected-area electron diffraction (SAED) pattern, in which regular separated diffraction spots also showed a well-crystalline structure. Furthermore, the extension direction of α-Si_3_N_4_ nanobelts coincided with the [101] crystal axis, indicating that the crystal growth direction of α-Si_3_N_4_ nanobelts is the [101] direction. In contrast to the clear α-Si_3_N_4_ nanobelts, β-Si_3_N_4_ were not observed under the HRTEM, even though the XRD result showed their existence.

### Surface energy calculation of three low-index crystallographic planes of Si_3_N_4_ nanobelts

**Figure 5:**
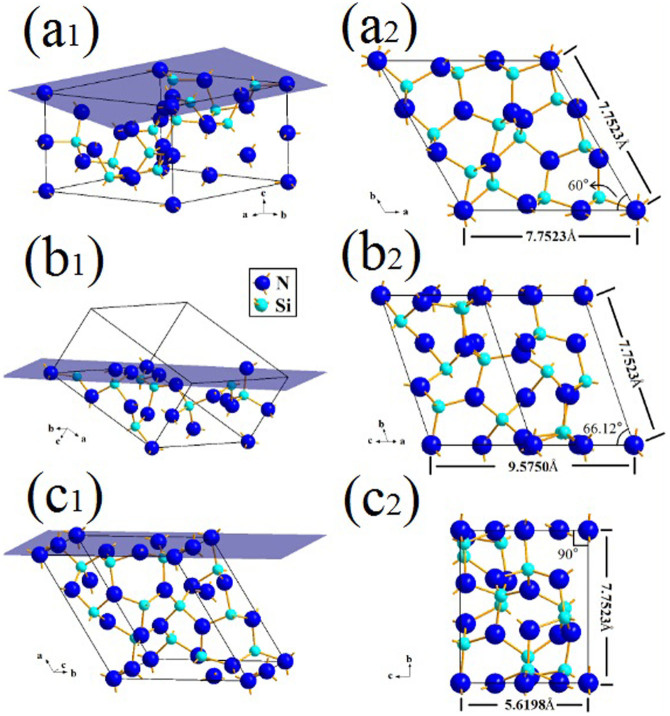
Unit cell model of α-Si_3_N_4_: (a1) (001), (b1) (101), (c1) (100). Projected view of lattice planes in the unit cell and lattice data: (a2) (001), (b2) (101), and (c2) (100).

It is believed that the morphologies of nanobelts are dominated by the combined effect of surface energy and growth kinetics^23^. In the previous studies on nanobelts, the number of broken bonds per unit area for each crystal plane was calculated to qualitatively compare the surface energy of the low-index planes^3^. In this study, the number of broken bonds for the (001), (101), and (100) planes in each unit cell were 10 (Fig. 5a1), 9 (Fig. 5b1), and 9 (Fig. 5c1), respectively. The projected view of the lattice planes in the unit cell and relative lattice data are shown in Figs. 5a2, 5b2, and 5c2. Accordingly, the number of broken bonds per unit area for each plane was calculated to be 0.19, 0.13, and 0.21 Å^−2^ for (001), (101), (100) planes, respectively. It can be concluded that the surface energy (δ) of three low-index planes follow the order: δ(100) > δ(001) > δ(101). The lower-energy planes can be easily the enclosure surfaces because of their smaller surface energies, whereas higher-energy planes constantly grow with the sufficient supply of raw materials. This indicates that nanobelts grow along the [100] plane, not the opposite experimental result of the [101] plane. The difference between the real growth direction and surface energy calculation shows that surface energy is not the dominating factor for Si_3_N_4_ nanobelt growth in this study. For the growth kinetics, the roles of factors such as growth temperature and flow stream parameters in determining the shape of nanobelts have not been clearly understood and need to be explored in the future.

### Combined mechanisms of vapor−liquid−solid (VLS) base growth and vapor–solid (VS) tip growth of Si_3_N_4_ nanobelts

**Figure 6:**
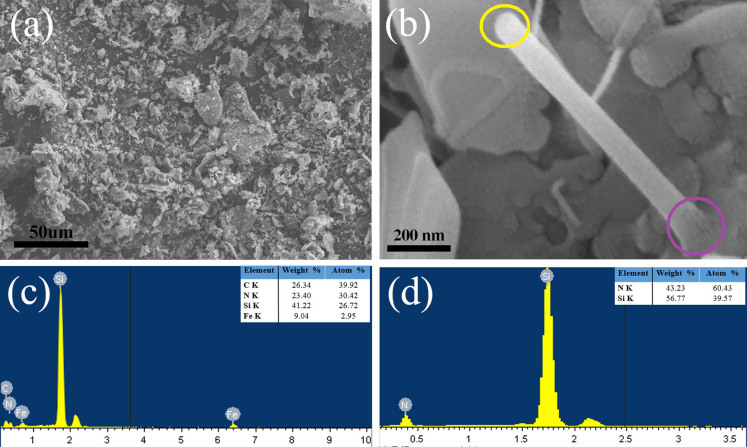
(a) SEM image of residual reactant in corundum crucible. (b) SEM image of initial growth stage of nanobelt. (c) EDS pattern at the bottom of the nanobelt marked by the purple ring in Fig. 6b. (d) EDS pattern at the tip of the nanobelt marked by the yellow ring in Fig. 6b.

**Figure 7:**
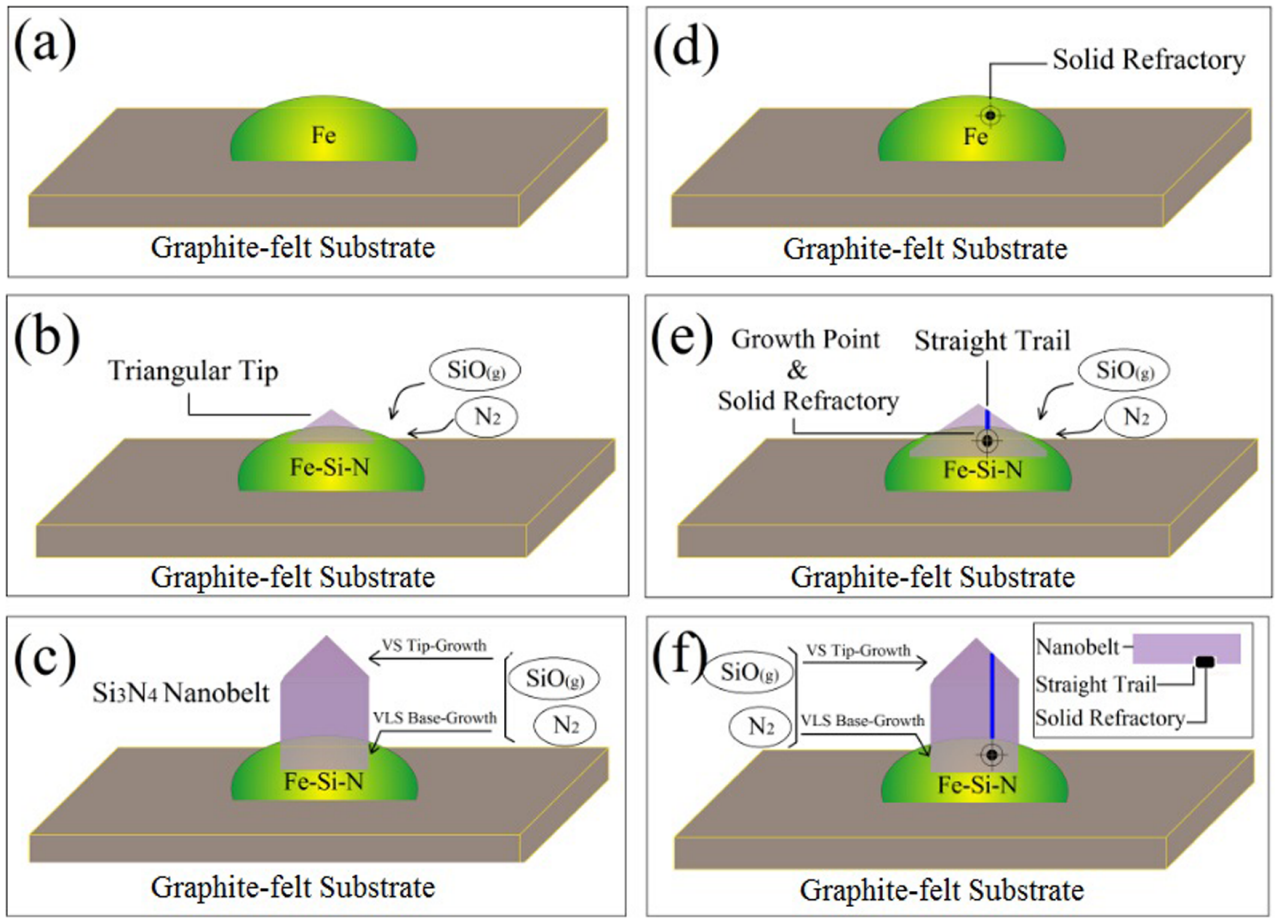
Proposed growth model of Si_3_N_4_ nanobelt. Normal topical Si_3_N_4_ nanobelt: (a) Fe droplet left on the surfaces of graphite felt substrate. (b) Fe reacted with both SiO and N_2_ to form Fe-Si-N eutectic liquid droplets and triangular tip of Si_3_N_4_ nanobelt began to show up. (c) Continual SiO and N_2_ were transported to the reaction site to form the Si_3_N_4_ along the growth direction. Si_3_N_4_ nanobelt with straight trail: (d) Fe droplet with a patch of solid refractory. (e) Nanobelt grew from Fe-Si-N eutectic droplets but it was hindered at a certain growth point by the solid refractory. (f) The Si_3_N_4_ nanobelt with straight trail continued to grow (the inset is the vertical sketch).

In this study, no metal catalyst droplets were observed on the tips of the nanobelts as shown in the SEM and TEM images, indicating that the growth of Si_3_N_4_ nanobelts did not follow the well-established VLS growth model^24^. Notably, Fe was detected at the bottom of Si_3_N_4_ nanobelts, whereas only Si and N were detected at the tip as shown in Figs. 6b, 6c, and 6d. The Si/N atomic ratios at the tip and bottom were 0.655:1 and 0.878:1, respectively. The result is slightly different than the stoichiometric ratio in Si_3_N_4_ (0.750:1), and various factors such as the scanning time, energy of X-ray, and amount and density of materials may be responsible for the accuracy of EDS spectrum^25^. The results of this and previous studies^15^,^21^ indicate that the VLS base growth and VS tip growth contribute to the growth of Si_3_N_4_ nanobelts. This process is described in detail as follows:

First, Fe(NO_3_)_3_ underwent thermal decomposition at a certain temperature to afford Fe_2_O_3_ (reaction 1). Then, Fe_2_O_3_ was reduced to Fe/FeO in the presence of C/CO (reactions 2 and 3), and the latter was left on the surface of graphite-felt substrate (Fig. 7a). It is generally accepted that the vapor phase of SiO plays an important intermediary role in CRN^26^,^27^,^28^. With the increase in temperature, SiO vapor was produced by the reaction of SiO_2_ with raw material graphite (reaction 4) and/or by the reaction of SiO_2_ with CO vapor (reaction 5). When the SiO vapors diffuse to the surface of graphite-felt substrate, the Fe droplets react with both SiO and N_2_ in a short time to form Fe–Si–N eutectic liquid droplets (Fig. 7b). It has been reported that Fe catalyst catalyzes the formation of these eutectic liquid droplets, which may have promoted the nucleation of Si_3_N_4_ and played a dominant role in the primary formation of belt-like morphology^15^. In this study, Fe (NO_3_)_3_ was added in the initial stage and decomposed at a certain temperature. Owing to the strong adhesion between the eutectics and substrate, the Fe catalyst was not observed at the tip of nanobelts, rather acted as the root for the continuous growth of nanobelts. Notably, the residual reactant in graphite crucible was observed as shown in Fig. 6a. No belt-like product was obtained in all the horizons, further confirming the importance of Fe(NO_3_)_3_ in the growth of Si_3_N_4_ nanobelts.

Based on the generated Fe–Si–N eutectic, SiO and N_2_ are transported continually to the reaction site to form Si_3_N_4_ along the growth direction (Fig. 7c). Until now, the VLS base growth was considered as the main mechanism. At this stage, the CO vapor phase, produced by reactions (4), (6), and (7), has been found to be of great importance in carbothermic synthesis experiments^29^,^30^,^31^. A comparison of the tip morphology shown in Fig. 6b with those shown in Figs. 3g and 3h and the larger size and smoother triangular tips indicated that the tips of Si_3_N_4_ nanobelts would grow in the late-stage reaction. Therefore, the VS tip-growth mechanism may play a role in the tip growth^21^,^22^, i.e., when the tip was grown from the Fe–Si–N eutectic, the SiO and N_2_ gas stacked at the nanobelt tip slowly but continuously, supporting the growth. Although the growth mechanism of the combined VLS growth and VS growth was discussed, the VLS growth mechanism is the dominating factor in the [101] direction. Thus, the Si_3_N_4_ nanobelts were gradually fabricated. The appearance of different phases can be attributed to the existence of low-melting eutectics, which were caused by metallic impurities (K_2_O, Na_2_O, TiO_2_, Al_2_O_3_, Fe_2_O_3_, and CaO) in the starting materials^32^,^33^. In this study, a small amount of metallic impurities (1.53% CaO, 0.11% Fe_2_O_3_, 0.07% Al_2_O_3_, and 0.01% K_2_O), which was introduced by the quartz raw material, was considered the main reason for the formation of β-Si_3_N_4_ crystals.
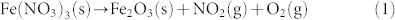

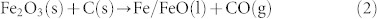

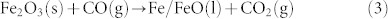

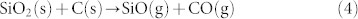

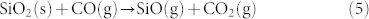

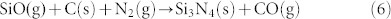

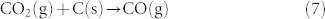


As mentioned above, the formation of a clear straight trail of several nanometers on the nanobelt (Fig. 3f) is difficult to understand because of its special location and great consistency. Based on the growth mechanism of Si_3_N_4_ nanobelts, the straight trail may have been guided by the Fe–Si–N eutectic liquid droplets. During the nucleation, a patch of solid refractory material was added to the eutectics and deposited at the growth point of nanobelts (Fig. 7d). With the supersaturation of the catalyst droplets, nanobelts grew from other points, but they were hindered at that point (Figs. 7e and 7f). The refractory material acted as a special template and guided the morphology of surface. As shown in Figs. 3c, 3d, and 3e, the straight trail is not located at the center of the nanobelts. In particular, Fig. 3c shows that a nanobelt is positioned well behind the curving nanobelt, and a sideling straight tail diagonally crosses the surface, but not at the center. We have observed many other nanobelts whose surface also have similar tails and found that the position of tails was uncertain; therefore, we believe that the position of a trail depends on the specific growth circumstance such as the initial position of solid refractory material as mentioned above and the direction of flow stream. Based on these evidences, the growth model is shown in Figs. 7d, 7e, and 7f, and the straight trail is shown at completely random places.

### Photoluminescence properties of as-prepared Si_3_N_4_ nanobelts

**Figure 8:**
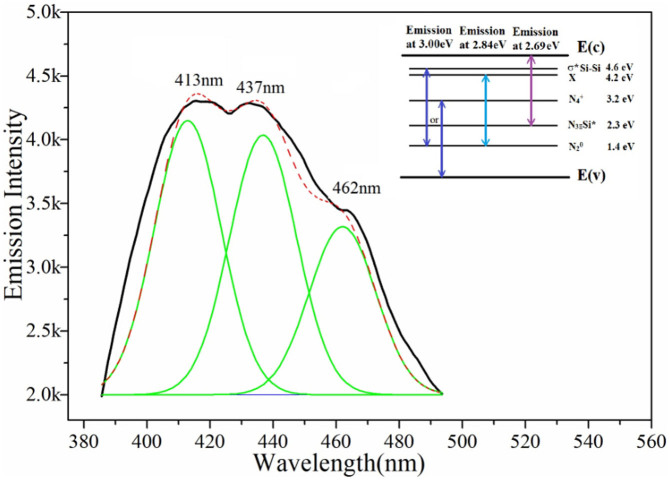
The emission spectra of Si_3_N_4_ nanobelts under 365 nm excitation (the black line is the as-obtained PL line, the red and green line is the simulated line). The top right corner is a simplified energy transitions between different energy levels.

Fig. 8 shows the room-temperature PL spectrum of the Si_3_N_4_ nanobelts measured at the excitation of 365 nm (3.40 eV). As shown in the spectrum, three emission peaks were observed in the violet–blue spectral range: 413 nm (3.00 eV), 437 nm (2.84 eV), and 462 nm (2.69 eV). A comparison of the direct band gap of Si_3_N_4_ (5.0–5.3 eV) shows that the three peaks clearly did not arise from the energy transition between the valence and conduction bands. They can be attributed to the trap-level defects in the materials. Normally, Si_3_N_4_ has a tetragonal structure where the Si atom is tetracoordinated by the N atoms, and the N atoms are tricoordinated by the Si atoms^34^. It has been widely accepted that four types of defects are present in Si_3_N_4_: Si–Si and N–N bonds, and Si and N dangling bonds^32^. Furthermore, a bonding σ orbital and an antibonding σ* orbital were formed by the Si–Si bond, and the energy gap was ~4.6 eV^36^,^37^. The dangling bonds also led to energy level splitting. The Si dangling bonds form one defect state in the midgap between these two orbitals marked as N_3_ ≡ Si*^35^,^38^. The other two N defective states are called N_4_^+^ and N_2_°, generating the levels within the gap. Moreover, an unclear X level found by Zhang et al. was close to the N3 ≡ Si* level^39^. Based on a previous study, the PL behavior of the Si_3_N_4_ nanobelts can be explained by the model. The emission at 3.00 eV can be attributed to the recombination between the Si–Si σ* level and the N_2_° level or between the N_4_^+^ level and the valence-band edge. The peak at 2.84 eV can be attributed to the recombination from the N_2_° level to the X level. Huang et al. also reported a similar emission peak at 2.87 eV; however, they assumed that the presence of oxygen, which introduced the N–Si–O defective state in the gap, caused the emission ~2.8 eV^21^. As shown in the PL spectrum, the contribution from the 2.69 eV peak is much lower than the others. Thus, the double-photon absorption excitation from the valence edge to N_3_ ≡ Si* and then to the conduction edge may lead to the emission^39^.

## Discussion

The large-scale synthesis of Si_3_N_4_ nanobelts from quartz and graphite was achieved on a graphite-felt substrate by catalyst-assisted CRN under a flow of N_2_ at a temperature of 1550°C. The modified CVD route, which separated the as-prepared nanobelts from the raw materials, avoided the pollution of production and simplified the purification procedure. The length of the Si_3_N_4_ nanobelts was ~4–5 mm, and their thickness was ~60 nm. The growth direction was along the [101] axis. Both the VLS base growth and VS tip growth were considered as the main mechanisms for the growth of Si_3_N_4_ nanobelts. Fe(NO_3_)_3_ played a crucial role in the initial stage of the formation of nanobelts, and the method that daubed both sides of the graphite-felt substrate with the catalyst improved the yield of the product. This study provides a low cost and large-scale method for Si_3_N_4_ nanobelts. Further, the intense violet–blue visible PL with three emission peaks at 413, 437, and 462 nm can be attributed to the defects in Si_3_N_4_, indicating potential applications in optoelectronic nanodevices.

## Methods

### Materials

The quartz raw material (chemical composition: SiO_2_ = 98.28%, CaO = 1.53%, Fe_2_O_3_ = 0.11%, Al_2_O_3_ = 0.07%, and K_2_O = 0.01%) was obtained from Lianyungang Changtong Silica Powder Co., Ltd.), and graphite powder (AR) was obtained from Tianjin Guangfu Fine Chemical Research Institute.

### Synthesis of Si_3_N_4_ nanobelts

The synthesis was conducted in a graphite crucible. Quartz and graphite powder were weighed in a molar ratio of 1:2 and thoroughly dry-mixed in a polyurethane jar using agate balls for 6 h (a ball-to-powder weight ratio of 2:1). Each graphite reactor was designed to load 4 g of the prepared materials. A piece of graphite felt was used as the substrate after dipping in a Fe(NO_3_)_3_/ethanol solution (0.04 M). The catalyst-dipped graphite-felt substrate was placed on the top of the crucible, ~1 cm away from the raw materials. Then, the entire reactor was moved into the center of a vacuum furnace. After evacuating the tube to 0.01 MPa, high-purity N_2_ (purity 99.99%) was introduced until the pressure reached 0.03 MPa. The furnace was first heated to 1000°C at a rate of 10°C/min, then heated to 1550°C at a rate of 2°C/min, and maintained at this temperature for 3 h. After cooling to room temperature, a layer of white product was found to have grown on the graphite-felt substrate; the product was collected for characterization.

### Characterization

The phases of the product was characterized by XRD (Bruker D8 Advance X-ray diffractometer, Germany) using Cu K_α1_ radiation (λ = 1.5406 Å) with a step of 0.02° (2θ) and a scanning rate of 4°/min. The existence of chemical bonds were proven by Fourier transform infrared spectroscopy (FT-IR; Nicolet IR100/200 spectrophotometer, USA) in the wavenumber range 1200–600 cm^−1^. The microstructure was investigated by field-emission FESEM (Hitachi S-4800, Japan), EDS (Horiba, UK), and TEM/HRTEM (FEI Tecnai G^2^ F20, USA, an accelerating voltage of 200 keV). The PL property was measured using a fluorescence spectrophotometer (Hitachi F-4600, Japan) at room temperature under a Xe lamp excitation.

## Author Contributions

S.Y.L., Z.H.H., J.T.H. and M.H.F. conceived and designed the experiments. S.Y.L. and H.T.L. carried out the experiments. S.Y.L., M.H.F., Y.G.L. and X.W.W. analyzed the data. All authors discussed the results. S.Y.L., Z.H.H. and H.P.J. wrote the paper. M.H.F. improved the English language.
